# Europe’s collective failure to address the refugee crisis

**DOI:** 10.1186/s40985-016-0015-6

**Published:** 2016-07-08

**Authors:** Bayard Roberts, Adrianna Murphy, Martin McKee

**Affiliations:** ECOHOST – The Centre for Health and Social Change, Faculty of Public Health and Policy, London School of Hygiene and Tropical Medicine, 15-17 Tavistock Place, London, UK

**Keywords:** Europe, European Union, Migrant, Refugee

## Abstract

The European response to the refugee crisis has been lamentable. A preoccupation with numbers has, too often, ignored how each refugee is an individual, many of whom have experienced the most appalling conditions in their countries of origin and in transit. These stories are only rarely heard, when the cameras are there to capture the tragedies. In this commentary we review the challenges of responding to the health needs of refugees, including examples of best practice, but above all call for a concerted political response that will both reduce the pressure on refugees to flee conflict-afflicted countries and recognize their contribution if they do come to Europe.

## Introduction: behind the numbers

“Il avait un nom (he had a name)” wrote Manuel Valls, the French Prime Minister, about the picture that will forever be associated with the current refugee crisis, a police officer on a Turkish beach picking up the body of the young Syrian boy Alan Kurdi. He reminded us how each of the almost a million refugees who crossed the Mediterranean into Europe in 2015, and the over 3000 who died trying, is a person with a story. Other images portray shipwrecks, people cut adrift in the sea, and facing humiliation at European borders or sheltering in squalor in camps such as those near Calais, in France. And these are only a fraction of the estimated 4 million or more registered refugees who have been displaced to countries neighbouring Syria, including 2.1 million in Turkey and 1.1 million in Lebanon [1].

Even when they reach comparative safety in Europe their plight continues. Humanitarian agencies are reporting that many refugees lack access to even basic primary health care, including maternal and child health services, and those with non-communicable diseases lack the continuity of care on which their health depends. Concerns have also been raised about the risk of sexual and gender-based violence, while children and young people are being separated from their families and left with limited protection. Europol has estimated that up to 10,000 unaccompanied child refugees have disappeared in Europe during the current crisis [2].

## The challenge … and the response

Providing health care for a population as it moves through Europe, being passed from one country to the next, has been immensely challenging. Governments in several countries have restricted even further the already limited entitlements of undocumented migrants [3–5]. The task of caring for them has fallen largely to civil society, involving small teams of volunteers that have sprung up in many European countries, as well as the longer established international humanitarian agencies such as Médecins du Monde, Médecins Sans Frontières, and Save the Children. They have provided medical and psychological support to refugees in ports, train stations, borders and transit camps using mobile clinics and fixed services [6]. Many statutory agencies have done little; Médecins du Monde had to take the local council in Calais to court because the conditions in the camp there were “a violation of their human rights, dignity and right to request asylum”, and where “dental abscesses and a scabies epidemic go untreated, and women are raped” [7]. Mental health is a particular problem, with many refugees experiencing traumas before, during and after migrating, and facing continued disadvantage in their new countries [6, 8].

Responding effectively is not easy. An effective response must overcome language barriers, impoverishment, lack of health insurance coverage, unfamiliarity with the health care system, different understandings of illness and treatment, distrust between staff and patients, and lack of access to the refugee’s medical history [9]. Yet there is much evidence of how to overcome these problems [10], including appropriate interpreting services (often available within the migrant community), cultural awareness by health workers, clear guidelines on entitlements of different migrant groups to care, and coordination between services [11, 12], but too often what is known to work is not done.

## The need for a political response

Important though these immediate responses are, they are no substitute for an effective political response, nationally and internationally. Many governments seem to have forgotten how, within living memory, their own citizens were fleeing oppression. For example, Hungarians, whose government has been among the most hostile to refugees, with its Prime Minister describing them as a threat to Europe’s Christian identity, were themselves welcomed in large numbers when they fled the Soviet invasion of their country in 1956. This view contrasts starkly with that of Pope Francis who memorably said “when the stranger in our midst appeals to us, we must not repeat the sins and the errors of the past” [13].

A few countries, such as Germany, have risen to the challenge but most have failed to implement their obligations under international law to protect refugees. The World Health Organization Regional Office for Europe and the European Centre on Disease Prevention and Control have done much, in areas such as monitoring and surveillance, delivery of medical supplies, and training of health workers, and have sought to combat public misconceptions on health and migration [14, 15]. However, both have only limited resources.

There is an obvious limit to what some of the smaller and poorer European countries can do alone, but the response by many of the richer ones has been begrudging and woefully inadequate. The United Kingdom is accepting just 20,000 Syrian refugees over a 5-year period, and even they will only be allowed to stay for 5 years, after which they will have to apply for and be granted asylum or be deported [16]. The EU’s 17-point plan agreed in October 2015 sought to increase cooperation, coordination, resources, and monitoring of the refugee response [17] but its implementation remains unclear, and long-term agreement on equitable distribution of refugees between the European Union member states remains elusive.

European engagement with the humanitarian needs of populations in their source and surrounding countries has also been inadequate. The United Nations is facing a funding crisis in its ability to care for the approximately 4 million registered refugees in the countries bordering Syria [1]. The WHO received only $5.1 million of the $60m it must raise for health care in Iraq. The head of the United Nations High Commission for Refugees has described the global humanitarian system as “financially broke,” [18] a key factor pushing many refugees to take the perilous journey to Europe. To its credit, the British government is the largest bilateral European funder of the Syrian refugee humanitarian response to these bordering countries but much greater contributions are required from it and from all the governments of Europe and other high-income countries.

An effective public health response must tackle the “causes of the causes”. In this case it means calling for action to tackle the fundamental problem, the conflicts in Syria and Iraq, to which those European countries now so reluctant to accept the resulting refugees contributed by their failures during and after the invasion of Iraq.

A re-framing of the refugee crisis is also required. David Cameron spoke of “a swarm of people coming across the Mediterranean” as if they were a pestilence rather than desperate men, women and children fleeing war and impoverishment [19]. The views that underpin a statement such as this are aired constantly in the tabloid press in some countries, taking every opportunity to exploit those cases where migrants do commit crimes or contravene established social norms, such as sexual harassment [20]. It is undeniable that such events do occur, and clearly should be condemned, but it is equally clear that some accounts are fabricated [21]. It should also be recalled that some of those newspapers that have been most vocal in their attacks on migrants used similar language about those fleeing the Third Reich in the 1930s. In one recent experiment, comments taken from actual statements made in the 1930s, including quotes from Mein Kampf, were posted on the website of a British newspaper, substituting “migrants” for “Jews” [22]. Many attracted hundreds of “likes” from readers.

Rather than pandering to xenophobia, Cameron and other politicians should join those who have shown leadership and recognize the social and economic benefits refugees can bring. They could do worse than emulate Angela Merkel, who deserves great credit for putting principles above short-term popularity. Perhaps her experience as the child of a minister of religion in the German Democratic Republic gave her some insight into what it means to be an outsider.

It is, however, also important to understand the reasons why those living in some countries are fearful of migrants. Where governments have allowed them to do so, unscrupulous employers have driven down wages and reduced job security, with some countries permitting so-called “zero hours” contracts where the employee has no guaranteed income. They have been able to do so in the knowledge that they can recruit migrants willing to accept these harsh conditions, thereby reducing employment opportunities for the existing population. Hence, at least part of the challenge of migration is how to balance the need by large businesses for cheap labour with the rights of all workers, a task made more difficult by attacks on trade unions in some countries. Unfortunately, as with the financial crisis, the interests of the former predominate.

Politicians should also join so many of their own citizens who have rescued refugees. Take Antonis Deligiorgis, a sergeant in the Greek army, who was pictured after diving into the sea to rescue Wegasi Nebiat [23]. She was a 24 year old Eritrean who, with 92 others, was on a makeshift boat that foundered on the rocks of Rhodes and, without his prompt action, would likely have drowned. And those who, less dramatically, but as importantly, have welcomed refugees into their homes, giving rise to the term “Wilkommenkultur”.

## Conclusions: a problem for refugees, and a problem for Europe

The refugee crisis that has confronted Europe has posed a major challenge to European structures. With a few notable exceptions, there has been an abject failure of political leadership. The Schengen Agreement, allowing free movement across borders, has been tested almost to destruction, as border controls are reinstated, barbed wire fences erected, and bridges are closed. The failure by nearly all of Europe’s leaders to take effective action, both to stabilise the countries from which migrants are coming, thereby reducing the pressure to move, and to make the positive case for migration in a continent experiencing a rapid decline in birth rate, has created a vacuum that xenophobic, racist, and neo-fascist politicians can occupy. This is a problem for those refugees who have fled to Europe, but is arguably an even greater problem for the citizens of Europe, who can now see that they are ruled by a political class that is incapable of acting for the common good of all of Europe. Europe has been here before, in the 1930s, but, tragically, our leaders seem incapable of learning the lessons of history.
